# Whole Exome Sequencing of Patients With Heritable and Idiopathic Pulmonary Arterial Hypertension in Central Taiwan

**DOI:** 10.3389/fcvm.2022.911649

**Published:** 2022-06-22

**Authors:** Kae-Woei Liang, Sheng-Kai Chang, Yu-Wei Chen, Wei-Wen Lin, Wan-Jane Tsai, Kuo-Yang Wang

**Affiliations:** ^1^Cardiovascular Center, Taichung Veterans General Hospital, Taichung, Taiwan; ^2^Institute of Clinical Medicine and Faculty of Medicine, School of Medicine, National Yang Ming Chiao Tung University, Taipei, Taiwan; ^3^School of Medicine and School of Life Science, National Chung Hsing University, Taichung, Taiwan; ^4^Excelsior Biopharma Inc., Taipei, Taiwan; ^5^Department of Life Science, Tunghai University, Taichung, Taiwan; ^6^Center for Pulmonary Arterial Hypertension and Pulmonary Vascular Disease, China Medical University Hospital, Taichung, Taiwan

**Keywords:** activin receptor-like kinase 1 (*ACVRL1*), aquaporin 1 (*AQP1*), bone morphogenetic protein receptor type-2 (*BMPR2*), pulmonary arterial hypertension (PAH), whole exome sequencing (WES), gene variants

## Abstract

**Background:**

Genetic variants could be identified in subjects with idiopathic and heritable pulmonary arterial hypertension (PAH). The 6th World Symposium on Pulmonary Hypertension (WSPH) provided a list of genes with evidence of association with PAH. However, reports using whole exome sequencing (WES) from southeastern Asian PAH cohorts were scarce.

**Methods:**

Subjects with idiopathic and heritable PAH (*N* = 45) from two medical centers in central Taiwan were screened for PAH related gene variants. The genomic DNA was prepared from peripheral blood lymphocytes. We performed WES for all patients enrolled in this study. All identified gene variants were validated by polymerase-chain reaction and Sanger sequencing. The clinical and hemodynamic data were compared between bone morphogenetic protein receptor type-2 (*BMPR2*) gene variants carriers vs. non-carriers.

**Results:**

Eight patients (8/45 = 17.8%) was identified carrying *BMPR2* gene variants and 8 patients (8/45 = 17.8%) had other WSPH-listed PAH-related gene variants (1 with *ACVRL1*, 1 with *ENG*, 1 with *SMAD9*, 1 with *SMAD1*, 1 with *ATP13A3* and 3 with *AQP1*). In addition, a total of 14 non-WSPH-listed PAH-related genetic variant sites (*ABCC8, NOTCH1, NOTCH2, NOTCH3, JAG1, BMP10, GGCX, FBLN2, ABCA3* and *PTGIS*) were found in this PAH cohort. Subjects carrying *BMPR2* gene variant (*N* = 8) were younger at diagnosis of PAH (30 ± 11 vs 49 ± 13 years, *p* = 0.001) than the non-carrier group (*N* = 37). *BMPR2* variant carriers had a trend toward having higher mean pulmonary arterial pressure (PAP) (61 ± 19 vs. 51 ± 13 mmHg, *p* = 0.076) than the non-carriers upon initial diagnosis. Pulmonary vascular resistance, right atrial pressure, cardiac output, as well as functional class were similar between *BMPR2* variant carriers and non-carriers at initial diagnosis.

**Conclusions:**

We identified 17.8% of patients with *BMPR2* gene variants and 17.8% subjects with other 6th WSPH-listed PAH-related gene variants in a Taiwanese idiopathic and heritable PAH cohort. PAH patients carrying *BMPR2* variants presented at a younger age with a trend toward having higher mean PAP at initial diagnosis.

## Introduction

It is now well established that around 70–80% of heritable pulmonary arterial hypertension (PAH) and 10–20% of idiopathic PAH cases are related with genetic variants in bone morphogenetic protein receptor type-2 (*BMPR2*) (1, 2). *BMPR2* deficiency leads to an abnormal over-activation of the transforming growth factor β (TGF-β) signaling pathway, resulting in over-proliferation of vascular smooth muscle cells at pulmonary arteriole (3).

Genetic variants in other components of the TGF-β/BMPR2 signal pathways, including activin receptor-like kinase 1 (*ACVRL1*) and endoglin (*ENG*) have been reported in PAH patients (2, 4–6). BMPR2 and ACVRL1 form a signaling complex, utilizing ENG as a co-receptor in endothelial cells of pulmonary arterioles (6). *ACVRL1* or *ENG* genetic variants have been found in patients with hereditary hemorrhagic telangiectasia (HHT) combined with PAH (7). Genetic variants downstream of BMPR2 signaling intermediaries, *SMAD1, SMAD4 and SMAD9* are also reported to be associated with PAH pathogenesis (6, 8).

Genes not directly impacting on the TGF-β/BMP pathway, including aquaporin 1 (*AQP1*), which encodes the plasma membrane water channel aquaporin 1, and ATPase family homolog up-regulated in senescence cells 1 *(ATP13A3*), an ATPases involved in ion channel transport, were reported to be associated with PAH (2, 6, 8). A distinct form of PAH, pulmonary veno-occlusive disease (PVOD) or pulmonary capillary hemangiomatosis (PCH), was shown recently to be caused by homozygous mutations in eukaryotic translation initiation factor 2 alpha kinase 4 (*EIF2AK4*), a kinase in the integrated stress response (6, 8–10). The 6^th^ World Symposium on Pulmonary Hypertension (WSPH) provided a list of pulmonary arterial hypertension genes with higher or lower level of evidence (2).

In addition to the genetic variants listed by WSPH (2), there are several new candidate genes with related reports for their associations with PAH. *ABCC8* (ATP-binding cassette, subfamily C, member 8) encodes SUR1 (sulfonylurea receptor 1), a regulatory subunit of the ATP-sensitive potassium channel (11). *ABCC8* loss-of function mutations in both pediatric and adult-onset PAH had been reported (11, 12). NOTCH signaling is involved in vascular morphogenesis and function (13). A mechanistic link between Notch3 signaling and PAH is supported by the observation that *NOTCH3* KO mice are protected from hypoxia-induced PAH and that PAH can be treated by a g-secretase inhibitor that inhibits the NOTCH pathway (14). Chida et al. (15) identified two novel missense mutations (G840E and T900P) of the *NOTCH3* gene in two cases of childhood idiopathic PAH.

Patients with PAH carrying *BMPR2* genetic variants usually presented at a younger age with higher mean pulmonary arterial pressure (PAP) or pulmonary vascular resistance (PVR) (1, 16, 17). Meta-analysis also showed worse clinical outcomes for death or transplantation in *BMPR2* mutants compared with non-mutants with PAH (1). A pediatric idiopathic PAH cohort from Beijing reported 50% primary PAH patients carried PAH-related genetic variants, including *BMPR2* (30.5%), *ACVRL1* (6.1%), 1 in *ENG* (1.2%) etc. and the variant group had worse clinical outcomes (18).

PAH patients with *BMPR2* genetic variants are trended to have earlier onset and worse clinical scenarios (1, 18, 19). Some PAH Asian cohorts had a higher rate for carrying PAH-related genetic variants than the Caucasian cohorts (18–20). However, reports using whole exome sequencing (WES) for identifying genetic variants from Southeasten Asian idiopathic and heritable PAH cohorts are still rare. The aim of the current research is using WES to identify PAH-related genetic variants and investigate their hemodynamic and clinical profiles in a Taiwanese idiopathic and heritable PAH cohort.

## Materials and Methods

### Patient Population

**Figure 1:**
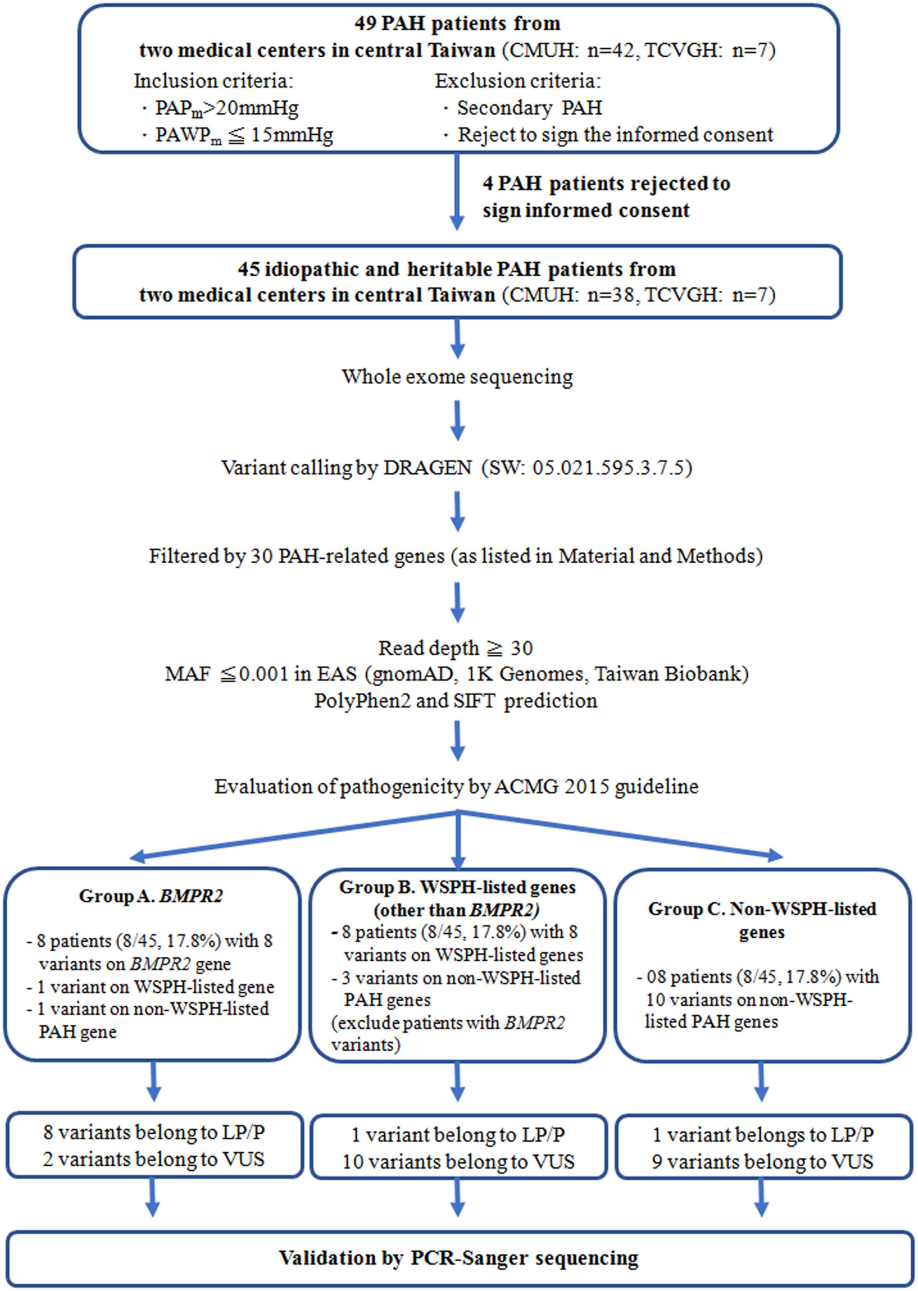
The study flow chart. PAH, pulmonary arterial hypertension; WSPH, The 6th World Symposium on Pulmonary Hypertension; *BMPR2*, bone morphogenetic protein receptor type-2; TCVGH, Taichung Veterans General Hospital (Taichung, Taiwan); CMUH, China Medical University Hospital (Taichung, Taiwan); ACMG, 2015 The American College of Medical Genetics and Genomics guidelines; P, pathogenic; LP, likely pathogenic; VUS, variant of uncertain significance; MAF, Minor allele frequency of East Asian, PAP_m_, mean pulmonary arterial pressure, PAWP_m_, mean pulmonary arterial wedge pressure; PCR, polymerase chain reaction.

We enrolled 45 idiopathic and heritable PAH patients from the Cardiology divisions of Taichung Veterans General Hospital (Taichung, Taiwan) and China Medical University Hospital (Taichung, Taiwan) for PAH genetic analysis between Mar. and Sep. 2021 (Figure 1). The diagnosis of PAH was based on the measurements of a mean PAP>20 mmHg at rest and a pulmonary arterial wedge pressure ≤ 15 mmHg by right heart catheterization (21). Secondary pulmonary hypertension was excluded by history, blood tests, chest computed tomography scan, nuclear ventilation-perfusion scan and echocardiography (21). We retrospectively collected information including age of initial PAH diagnosis, functional class at diagnosis, and medications and clinical worsening events after initial PAH diagnosis. This study was reviewed and approved by the Institutional Review Board of Taichung Veterans General Hospital (Approval Number, SE21052A) and China Medical University Hospital (Approval Number, CMUH109-REC3-198) (Taichung, Taiwan) and all patients gave their written consent to participate.

### Hemodynamic Measurements

All diagnoses were confirmed by right heart catheterization to measure hemodynamic values for all patients at their initial diagnosis (21). The mean PAP, pulmonary arterial wedge pressure, cardiac output, and PVR were recorded by standard techniques (21). The date of 1st right heart catheterization was deemed as the time of initial PAH diagnosis.

### Diagnosis Criterion for Hereditary Hemorrhagic Telangiectasia

The diagnosis is based on the Curacao criteria. These criteria include (1) recurrent and spontaneous epistaxis; (2) visceral localization (gastrointestinal telangiectasia, pulmonary, hepatic, cerebral or spinal arteriovenous malformations) (3) an affected first-degree family member; and (4) the presence of mucocutaneous telangiectases. When an individual shows three or more criteria, they are considered to have HHT. When they meet two criteria the diagnosis is possible and with one or none criteria, HHT is considered unlikely (7, 22).

### DNA Extraction, Whole Exome Sequencing and Data Analysis

Total genomic DNA was isolated from peripheral blood using NucleoSpin^®^ Blood Kit (Macherey-Nagel, Duren, Germany) according to manufacturer protocols. We performed the WES for all patients enrolled in this study. Whole exome libraries were processed using KAPA HyperExome Plus Kit, KAPA Universal Adapter, and KAPA HyperCapture Bead Kit according to the KAPA HyperCap Workflow v3.0 Workflow (Roche Sequencing and Life Science, MA, USA). The paired-end 2 × 150 bp sequencing was performed on the Illumina NovaSeq 6000 Sequencer (Illumina Inc., USA). For single nucleotide variants (SNV), the sequencing raw data was processed by DRAGEN platform (SW: 05.021.595.3.7.5) (Illumina Inc., USA). All obtained variants from Variant Call Format (VCF) file were further filtered by a panel of PAH-related genes including 16 WSPH-listed [*BMPR2, KCNK3, EIF2AK4, KLF2, ACVRL1, SMAD1, AQP1, SMAD4, ATP13A3, SMAD9, TBX4, SOX17, CAV1, KCNA5, BMPR1B, GDF2*(23) and *ENG*] (2) and 14 non-WSPH-listed genes [*ABCA3*(18, 24)*, NOTCH1*(25, 26)*, NOTCH2*(27, 28)*, NOTCH3*(15, 29)*, ABCC8*(11, 12)*, BMP10*(30, 31)*, FBLN2*(32)*, JAG1*(33)*, PTGIS*(34)*, PDGFD*(32)*, GGCX*(35)*, SMAD5*(36, 37)*, KLK1*(35) and *TopBP1*(26, 38)]. Those variants with read depth <30x were filtered out. The coverage of the specific gene was analyzed using WES Binary Alignment/Map (BAM) files. In this study, the average coverages of all candidate genes have >100×. All variants on those genes were compared with widely used normal subject control databases (gnomAD database, ExAC, 1000 Genomes Project and Taiwan Biobank) to filter those variants allelic frequencies more than 0.1% in East Asian group (EAS). The Polyphen2 and SIFT *in silico* software were applied to predict the impact of variants on specific genes. For structure variations on *BMPR2* gene, we applied ExomeDepth from a free R package (version 1.1.15) to detect CNVs on the *BMPR2* gene of all PAH patients (39). The suspected CNVs on *BMPR2* from ExomeDepth analysis software were subjected to validation through multiplex ligation-dependent probe amplification technology using SALSA MLPA Probemix P093 HHT/HPAH kit (MRC-Holland, Netherlands). The evaluation of clinical impacts followed the 2015 ACMG guidelines (The American College of Medical Genetics and Genomics) (40).

### Polymerase-Chain Reaction and Sanger Sequencing

All filtered variants identified from candidate genes were validated by polymerase-chain reaction (PCR) and Sanger sequencing. The specific PCR primers were designed manually to cover entire exons of genes. The PCR was conducted using PCRBIO Ultra Polymerase (PCR Biosystems, London, UK) and SensoQuest Labcycler (SensoQuest, Göttingen, Germany). The purified PCR products were directly sequenced with an ABI PRISM terminator cycle sequencing kit v3.1 on the ABI 3730 DNA sequencer (Applied Biosystems, CA, USA).

### Statistical Analysis

Continuous variables are expressed as mean ± SD and categorical data as percentages. The study subjects were grouped as *BMPR2* genetic variant carriers and non-carriers. Non-normally distributed continuous variables were compared with the Mann-Whitney U test, while normally distributed variables were compared with Student's t test. Categorical variables were compared by the Chi-square test with Yate's correction or Fisher's exact test. A two-tailed *p*-value < 0.05 was considered statistically significant. The SPSS 12.1 statistical software package (SPSS, Inc., Chicago, IL, USA) was used for all calculations.

## Results

### Details of *BMPR2* Gene Variants

A total of 45 idiopathic and heritable PAH patients were enrolled for analysis. We found *BMPR2* gene variants in 17.8 % (8/45) of PAH patients in central Taiwan (Table 1 and Figure 1). Among the 8 patients with *BMPR2* gene variants, 3 had frameshifts, 2 had non-sense, 2 had missense variants and 1 had *BMPR2* exon 8-9 deletions (Table 1 and Supplementary Figure 1). Among the 8 *BPMPR2* variants, 4 variant genetic types were previously reported in the literature (4, 5, 41–45). We identified three novel *BMPR2* gene variants (c.1512 deletion, c.479 duplicate, c.1165 G>A) in the current study and one patient with exon 8-9 deletions (Table 1). The patient (A365) with “c.1165 G>A” novel variant (resulting in missense amino acid change of Glu389Lys) presented with heritable PAH and a large pulmonary arteriovenous malformation (Table 2). However, she did not fit the diagnosis criterion for HHT (22).

**Table 1:** Details of genetic variants in PAH-related genes.

**No**.	**ID**	**Gender**	**WSPH genes**	**Gene**		**cDNA change**	**Amino acid change**	**Variant type**	**Genotype**		**ACMG**	**MAF in EAS**	**Ref**
										**PolyPhen2/SIFT**	**2015**		
**Group A. PAH patients with** ***BMPR2*** **variants (*****N*** **= 8)**													
**1**	A213	F	Y	*BMPR2*	NM_001204.7	c.1512del	p.Leu504Leufs*2	Frameshift	Het	NA/NA^$^	LP	0.00000	-
**2**	A255	F	Y	*BMPR2*	NM_001204.7	c.338dup	p.Tyr113*	Nonsense	Het	NA/NA^$^	P	0.00000	(4, 5, 41)
			Y	*SMAD9*	NM_001127217.3	c.767C>T	p.Ser256Leu	Missense	Het	B/D	VUS	0.00009	-
**3**	A256	F	Y	*BMPR2*	NM_001204.7	c.479dup	p.Leu161Ilefs*20	Frameshift	Het	NA/NA^$^	LP	0.00000	-
**4**	A258	F	Y	*BMPR2*	NM_001204.7	c.961C>T	p.Arg321*	Nonsense	Het	NA/NA^$^	P	0.00000	(42–44)
**5**	A365	F	Y	*BMPR2*	NM_001204.7	c.1165G>A	p.Glu389Lys	Missense	Het	PD/D	LP	0.00000	-
**6**	A387	M	Y	*BMPR2*	NM_001204.7	c.937G>C	p.Ala313Pro	Missense	Het	PD/D	LP	0.00000	(45)
**7**	A441	M	Y	*BMPR2*	NM_001204.7	c.1376_1377del	p.Arg459Thrfs*11	Frameshift	Het	NA/NA^$^	P	0.00000	(5, 46)
**8**	A450	M	Y	*BMPR2*	NM_001204.7	Ex8-9del	Unknown	Gross deletion	Het	NA/NA^$^	P	0.00000	
			N	*NOTCH3*	NM_000435.3	c.2183G>A	p.Arg728His	Missense	Het	B/T	VUS	0.00000	-
**Group B. PAH patients with variants in WSPH-listed related genes (*****N*** **= 8)**													
**9**	A001	F	Y	*AQP1*	NM_001329872.2	c.273C>G	p.Ile91Met	Missense	Het	B/D	VUS	0.00055	-
			N	*NOTCH3*	NM_000435.3	c.2299C>T	p.Arg767Cys	Missense	Het	PD/T	VUS	0.00036	-
**10**	A249	F	Y	*ACVRL1*	NM_000020.3	c.1293T>A	p.Asn431Lys	Missense	Het	PD/D	VUS	0.00019	-
**11**	A363	F	Y	*SMAD9*	NM_001127217.3	c.313C>G	p.His105Asp	Missense	Het	PD/D	VUS	0.00005	(36)-
**12**	A364	F	Y	*ENG*	NM_000118.3	c.694_706dup	p.Val236Alafs*102	Frameshift	Het	NA/NA^$^	LP	0.00000	-
		F	N	*JAG1*	NM_000214.3	c.1702C>T	p.Arg568Cys	Missense	Het	PD/T	VUS	0.00005	-
**13**	A423	F	Y	*ATP13A3*	NM_001367549.1	c.139_141del	p.Leu47del	Inframe deletion	Het	NA/NA^$^	VUS	0.00000	-
			N	*NOTCH1*	NM_017617.5	c.5422G>A	p.Asp1808Asn	Missense	Het	PD/T	VUS	0.00028	-
**14**	A431	F	Y	*AQP1*	NM_001329872.2	c.457G>A	p.Val153Met	Missense	Het	PD/D	VUS	0.00038	-
**15**	A492	F	Y	*SMAD1*	NM_001003688.1	c.68G>C	p.Gly23Ala	Missense	Het	PD/D	VUS	0.00058	-
**16**	A515	F	Y	*AQP1*	NM_001329872.2	c.968C>T	p.Pro323Leu	Missense	Het	NA/NA^$^	VUS	0.00000	-
**Group C. PAH patients with variants in non-WSPH-listed genes (*****N*** **= 8)**													
**17**	A214	F	N	*ABCC8*	NM_000352.6	c.206dup	p.Gly70Trpfs*19	Frameshift	Het	NA/NA^$^	LP	0.00000	-
			N	*PTGIS*	NM_000961.4	c.419T>C	p.Met140Thr	Missense	Het	PD/T	VUS	0.00071	-
**18**	A228	F	N	*NOTCH2*	NM_001200001.2	c.544G>A	p.Asp182Asn	Missense	Het	PD/D	VUS	0.00005	-
**19**	A229	F	N	*ABCA3*	NM_001089.3	c.1327G>A	p.Val443Met	Missense	Het	PD/T	VUS	0.00065	-
**20**	A230	F	N	*GGCX*	NM_000821.7	c.1579A>G	p.Thr527Ala	Missense	Het	PD/D	VUS	0.00005	-
**21**	A253	F	N	*JAG1*	NM_000214.3	c.3577C>T	p.His1193Tyr	Missense	Het	PD/T	VUS	0.00033	-
**22**	A397	F	N	*FBLN2*	NM_001004019.2	c.256G>A	p.Gly86Ser	Missense	Het	PD/D	VUS	0.00050	-
			N	*NOTCH2*	NM_001200001.2	c.6284A>G	p.Lys2095Arg	Missense	Het	B/T	VUS	0.00005	-
**23**	A413	F	N	*BMP10*	NM_014482.3	c.350C>T	p.Pro117Leu	Missense	Het	PD/D	VUS	0.00011	-
**24**	A476	M	N	*GGCX*	NM_000821.7	c.1985G>A	p.Arg662His	Missense	Het	PD/T	VUS	0.00016	-

*PAH, pulmonary arterial hypertension; WSPH, The 6th World Symposium on Pulmonary Hypertension; ACMG, 2015 The American College of Medical Genetics and Genomics guidelines; P, pathogenic; LP, Likely pathogenic; VUS, variant of uncertain significance. MAF, Minor allele frequency of East Asian in gnomAD exome databases*.

*Reference Sequences, BMPR2 (NM_001204.7), SMAD9 (NM_001127217.3), NOTCH3 (NM_000435.3), AQP1 (NM_001329872.2), ACVRL1 (NM_000020.3), ENG (NM_000118.3), JAG1 (NM_000214.3), ATP13A3 (NM_001367549.1), NOTCH1 (NM_017617.5), SMAD1 (NM_001003688.1), ABCC8 (NM_000352.6), PTGIS (NM_000961.4), NOTCH2 (NM_001200001.2), ABCA3 (NM_001089.3), GGCX (NM_000821.7), FBLN2 (NM_001004019.2), BMP10 (NM_014482.3)*.

*^$^PolyPhen2 and SIFT in silico prediction program were unable to analyze frameshift, nonsense, in frame deletion, gross deletion types and some variants on AQP1 gene with unknown reasons. PolyPhen2, PD, Probably damaging; D, Damaging; B, Benign. SIFT, D, Deleterious; T, Tolerated*.

**Table 2:** Pulmonary arterial hypertension patients with genetic variants and their clinical findings.

**No**.	**ID**	**Gender**	**Age**	**Gene**	**Nucleotide change (cDNA)**	**Amino acid change**	**Clinical findings**	**PAPm (mmHg)**	**PVR (WU)**	**CO (L/min/m^**2**^)**	**MVO_**2**_ (%)**
**Group A. PAH patients with** ***BMPR2*** **genetic variants (*****N =*** **8)**											
1	A213	F	45	*BMPR2*	c.1512del	p.Leu504Leufs*2	PAf	54	8	4.9	nil
2	A255	F	18	*BMPR2*	c.338dup	p.Tyr113*		89	25	3.3	58
				*SMAD9*	c.767C>T	p.Ser256Leu					
3	A256	F	39	*BMPR2*	c.479dup	p.Leu161Ilefs*20	Heritable PAH, elder brother with PAH	70	25.1	2.4	53.7
4	A258	F	35	*BMPR2*	c.961C>T	p.Arg321*		55	15	2.4	61
5	A365	F	38	*BMPR2*	c.1165G>A	p.Glu389Lys	The patient has PAVM, s/p occluder, fraternal aunt with PAH	34	10.3	2.4	61
6	A387	M	32	*BMPR2*	c.937G>C	p.Ala313Pro		73	12.3	5.2	70
7	A441	M	19	*BMPR2*	c.1376_1377del	p.Arg459Thrfs*11	Asthma	77	21.6	3.1	64
8	A450	M	17	*BMPR2*	Ex8-9del	Unknown		37	6	6.1	75.9
				*NOTCH3*	c.2183G>A	p.Arg728His					
**Group B. PAH patients with variants in WSPH- listed genes (*****N =*** **8)(other than** ***BMPR2*** **variants)**											
9	A001	F	37	*AQP1*	c.273C>G	p.Ile91Met	Heritable PAH, younger brother has PAH	57	20	2.1	46
				*NOTCH3*	c.2299C>T	p.Arg767Cys					
10	A249	F	29	*ACVRL1*	c.1293 T>A	p.Asn431Lys		59	21	2.4	56
11	A363	F	63	*SMAD9*	c.313 C>G	p.His105Asp		27	3	4.6	74.5
12	A364	F	82	*ENG*	c.694_706dup	p.Val236Alafs*102	PA aneurysm (5 cm)	60	15	3.0	62
				*JAG1*	c.1702C>T	p.Arg568Cys					
13	A423	F	37	*ATP13A3*	c.139_141del	p.Leu47del	PA aneurysm (8 cm) with LM coronary compression, s/p stenting	74	21.8	2.9	63
				*NOTCH1*	c.5422G>A	p.Asp1808Asn					
14	A431	F	35	*AQP1*	c.457G>A	p.Val153Met		45	9.3	3.2	61
15	A492	F	41	*SMAD1*	c.68G>C	p.Gly23Ala	PAf	56	20.1	Nil	nil
16	A515	F	55	*AQP1*	c.968C>T	p.Pro323Leu		67	21.2	2.7	59
**Group C. PAH patients with variants in non-WSPH-listed genes (*****N =*** **8)**											
17	A214	F	31	*ABCC8*	c.206dup	p.Gly70Trpfs*19		35	8.0	4.1	73.4
				*PTGIS*	c.419T>C	p.Met140Thr					
18	A228	F	58	*NOTCH2*	c.544G>A	p.Asp182Asn	PA Aneurysm (7.5 cm)	73	12.3	4.5	70
19	A229	F	43	*ABCA3*	c.1327G>A	p.Val443Met		47	6.5	4.4	72.8
20	A230	F	58	*GGCX*	c.1579A>G	p.Thr527Ala		39	7.4	4.0	76.2
21	A253	F	38	*JAG1*	c.3577C>T	p.His1193Tyr		44	11.4	3.4	78
22	A397	F	44	*FBLN2*	c.256G>A	p.Gly86Ser		64	17.5	3.1	60
				*NOTCH2*	c.6284A>G	p.Lys2095Arg					
23	A413	F	42	*BMP10*	c.350C>T	p.Pro117Leu		76	58.6	1.6	26.1
24	A476	M	45	*GGCX*	c.1985G>A	p.Arg662His		69	10.7	5.8	80

*CO, cardiac output (L/min); MVO2, mixed venous oxygen saturation (%); PAP_m_, mean pulmonary arterial pressure (mmHg); PVR, pulmonary vascular resistance (Wood unit); PA, pulmonary artery; PAH, pulmonary arterial hypertension; PAf, paroxysmal atrial fibrillation; PAVM, pulmonary arteriovenous malformation; LM, left main coronary artery; WSPH, The 6th World Symposium on Pulmonary Hypertension*.

### Details of WSPH-Listed PAH-Related Gene (Other Than *BMPR2*) Variants

The 6th WSPH provided a list of pulmonary arterial hypertension genes with higher or lower level of evidence (2). In this study, 8 patients (8/45 = 17.8%) had WSPH-listed PAH-related gene (other than *BMPR2*) variants (1 with *ACVRL1*, 1 with *ENG*, 1 with *SMAD9*, 1 with *SMAD1*, 1 with *ATP13A3* and 3 with *AQP1*) (Table 1 and Figure 1). All the mis-sense variant effects were checked by software PolyPhen2 and SIFT and were listed in Table 1. The patient (A249) with *ACVRL1* variant and the patient (A364) with *ENG* variant did not show clinical evidence of HHT (Table 2) (22).

### Details of Non-WSPH-Listed PAH-Related Gene Variants

Among the study cohort, we totally identified 14 genetic variants sites, which are not listed in WSPH PAH-related genes, from 12 patients (Figure 1 and Table 1). Novel genetic variants related with Notch pathway, such as *NOTCH3, NOTCH2, NOTCH1*, and *JAG1* were identified in this cohort, too (Table 1). One patient carried an *ABCC8* genetic variant, which is deemed likely pathogenic (Table 1).

### Family History of PAH in the Study Cohort

Among the 45 study subjects, 3 of them had family aggregate of heritable PAH (Table 2). Patient (A256) with *BMPR2* variant of c.479dup had a family history heritable PAH with her younger brother (not included in this study, data not shown) diagnosed with PAH, too. Patient (A365) with *BMPR2* variant of c.1165 G>A had a family history heritable PAH with her fraternal aunt (not included in this study, data not shown) diagnosed with PAH and carried the same *BMPR2* variant site. Though she had a large pulmonary arteriovenous malformation, she or her aunt did not fit the diagnosis of HHT. Her two siblings did not have PAH and did not receive genetic tests. Patient (A1) with *AQP1* variant of c.273C>G had a family history heritable PAH with her younger brother having PAH and carried the same *AQP1* variant site (not included in this study, data not shown) (Table 2).

### Clinical and Hemodynamic Data in Idiopathic and Heritable PAH Patients With or Without *BMPR2* Gene Variants

A total of 45 idiopathic and heritable PAH patients were enrolled for analysis (Table 3). Subjects carrying *BMPR2* gene variant (*N* = 8) were younger at diagnosis of PAH (30 ± 11 vs. 49 ± 13 years, *p* = 0.001) than the non-*BMPR2* variant group (*N* = 37). *BMPR2* variant carriers had a trend toward having higher mean PAP (61 ± 19 vs. 51 ± 13 mmHg, *p* = 0.076) than the non-carriers upon initial diagnosis. PVR, right atrial pressure, cardiac output, as well as functional class were similar between *BMPR2* variant carriers and non-carriers at initial diagnosis.

**Table 3:** Clinical and hemodynamic presentations at initial diagnosis for pulmonary arterial hypertension patients with or without *BMPR2* genetic variant.

**Characteristics**	**All (*N =* 45)**	***BMPR2*** **gene variant carrier** **(*N =* 8)**	**Non-*BMPR2* variant carrier** **(*N =* 37)**	***p*** **-value**
Age at diagnosis (yr)	46 ± 15	30 ± 11	49 ± 13	0.001
Male/Female	9/36	3/5	6/31	0.380
PAP_m_ (mmHg)	53 ± 15	61 ± 19	51 ± 13	0.076
PAWP_m_ (mmHg)	12 ± 4	10 ± 2	12 ± 5	0.335
PVR (Wood unit)	14 ± 10	15 ± 8	13 ± 10	0.565
RAP_m_ (mmHg)	10 ± 5	11 ± 3	10 ± 5	0.473
CO (L/min)	3.5 ± 1.2	3.7 ± 1.5	3.4 ± 1.1	0.490
TR PSPG (mmHg)	84 ± 28	89 ± 33	82 ± 28	0.528
NT-proBNP (pg/ml)	1,741 ± 2,275	1,974 ± 1,291	1,691 ± 2,445	0.770
Functional class				0.073
I	1	1	0	
II	1	0	1	
III	34	7	27	
IV	9	0	9	

*BMPR2, bone morphogenetic protein receptor 2; CO, cardiac output (L/min); NT-proBNP, N-terminal pro-B type natriuretic peptide; PAP_m_, mean pulmonary arterial pressure (mmHg); PVR, pulmonary vascular resistance (Wood unit); PAWP_m_, mean pulmonary arterial wedge pressure (mmHg); RAP_m_, mean right atrial pressure (mmHg); TR PSPG, echocardiographic derived tricuspid valve regurgitation peak systolic pressure gradient*.

## Discussions

Idiopathic and heritable PAH patients with genetic variants are usually younger with worse hemodynamic or clinical profiles in Caucasians and Northeastern Asian cohorts (1, 18). However, reports from southeastern Asian cohorts are scarce. In this study, we identified 17.8% of patients with *BMPR2* gene variants and 17.8% subjects with WSPH-listed PAH-related gene (other than *BMPR2*) variants in a Taiwanese PAH cohort. Idiopathic and heritable PAH patients carrying *BMPR2* variant presented at a younger age with a trend toward having higher mean PAP at initial diagnosis.

*BMPR2* genetic variants will lead to receptor protein deficiency and result in abnormal overactivation of the TGF-β1 signaling pathway (3). The TGF-β pathway over-activation leads to proliferation of vascular smooth muscle cells and increase of PVR in pulmonary circulation in primary PAH (3, 46). Asian cohort from Korea reported that the prevalence of *BMPR2* variants in Korean primary PAH patients was 22% (19). A Japanese group reported *BMPR2* variants in all 4 familial (4/4 = 100%) and 12 (12/30 = 40%) of the sporadic PAH cases, which was higher than the proportions reported in Caucasian patients (20). Another Japanese group demonstrated that with state-of-the-art therapy, the long-term survival of patients with PAH was high, regardless of the *BMPR2* mutation status (47). Evans et al. conducted a meta-analysis of 1,550 patients with idiopathic, heritable, and anorexigen-associated PAH from eight cohorts and showed that 29% had *BMPR2* gene mutations (1). In our study from central Taiwan, we identified *BMPR2* variants in 17.8 % (8/45) of primary PAH patients, which is somewhat lower than previously reported prevalence from other cohorts. However, we identified three novel *BMPR2* gene variants (c.1512 deletion, c.479 duplicate, c.1165 G>A) in the current study (Table 1), which were not reported in literature before.

The 6th WSPH listed *BMPR2, EIF2AK4, TBX4, ATP13A3, GDF2, SOX17, AQP1, ACVRL1, SMAD9, ENG, KCNK3* and *CAV1* mutations as higher level of evidence and *SMAD4, SMAD1, KLF2, BMPR1B* and *KCNA5* with lower level of evidence of association with primary PAH (2). Mutations of *ACVRL1, ENG, SMAD1, SMAD4*, and *SMAD9* are closely related with alterations of the TGF-β/BMPR2 signal pathways in pathogenesis of primary PAH (2, 4–6). In this study, we used WES and identified 17.8% subjects with 6th WSPH listed PAH-related gene variants (other than *BMPR2*) in a Taiwanese idiopathic and heritable PAH cohort. The protein product of *EIF2AK4* belongs to a family of kinases that regulates angiogenesis. Specifically, these kinases phosphorylate the α subunit of eukaryotic translation initiation factor 2, a protein that downregulates protein synthesis in response to varied cellular stresses (9). In this study, none of our study subjects had homozygous *EIF2AK4* variants and nor did anyone present with evidence of PVOD or PCH (9, 10, 48). Three of study patients had *EIF2AK4* heterozygous variants (Supplementary Table 1) (48). The mutations of *ACVRL1*, a TGF-β/BMPR2 signal pathway member, have been found to be associated with PAH. In addition, *ACVRL1* mutation has been found in patients with HHT combined with PAH (7). One study subject (A249) carried *ACVRL1* variant but did not show clinical evidence of HHT. Previous report from Zhang et al. (49) reported primary PAH patients with *ACVRL1* mutation have rapid disease progression, high overall mortality rate and no response to the acute pulmonary vasodilation test.

In terms of non-WSPH-listed PAH related genes, we identified 14 novel sites in 12 patients (Table 1). *ABCC8* encodes SUR1, a regulatory subunit of the ATP-sensitive potassium channel (11). *ABCC8* loss-of function mutations in both pediatric and adult-onset PAH had been reported (11, 12). We identified a genetic variant of c.206dup, which is deemed likely pathogenic. Notch receptors, several ligands, and components of the downstream signaling machinery are expressed in the vessel and have been reported to be related with PAH (13, 14, 50). We identified several novel genetic variant sites in *NOTCH1, NOTCH 2, NOTCH 3* and *JAG1* in this PAH study cohort (Table 1). However, we did not conduct further *in vitro* studies to prove their genetic impacts on the downstream cell signals.

This study had several limitations. First, the PAH patients were recruited from two medical centers in central Taiwan. Thus, the patient numbers were limited. Second, the PAH patients were enrolled for genetic analysis in a specific time frame between Mar. and Sep. 2021. Their clinical and hemodynamic data were then retrospectively traced back to their initial diagnosis date. Those who had the worst clinical scenario with rapid disease progression to death had no chance to be retrospectively enrolled in this genetic study. Third, we identified several novel variant sites for *BMPR2* as well as other WSPH-listed or non-listed PAH-related genes. We only used software PolyPhen2 and SIFT to exam all the mis-sense mutations but did not have further *in vivo* studies to confirm that the genetic variants did have functional changes to the downstream proteins.

In conclusion, we identified 17.8% of patients with *BMPR2* gene variants and 17.8% subjects with other WSPH-listed PAH-related gene variants in a Taiwanese idiopathic and heritable PAH cohort. In addition, we also identified 14 none WSPH-listed PAH-related genetic variants sites from 12 patient. PAH patients carrying *BMPR2* variants presented at a younger age with a trend toward having higher mean PAP at initial diagnosis.

## Data Availability Statement

The datasets presented in this study can be found in online repositories (NCBI BioProject Accession: PRJNA824211).

## Ethics Statement

The studies involving human participants were reviewed and approved by Taichung Veterans General Hospital, China Medical University Hospital (Taichung, Taiwan). The patients/participants provided their written informed consent to participate in this study.

## Author Contributions

K-WL wrote the manuscript and analyzed/interpreted the data. S-KC performed the laboratory work. K-YW, K-WL, W-WL, W-JT, and Y-WC recruited the patients and critically reviewed the manuscript for important intellectual content. K-YW designed the study. All authors read and approved the final manuscript.

## Funding

The whole exome sequencing, polymerase-chain reaction and Sanger sequencing of the genomic DNA were sponsored by Excelsior Biopharma Inc. and conducted at its Genetic Laboratory. The author S-KC is employed by Excelsior Biopharma Inc. The Excelsior Biopharma Inc. was not involved in the study design, collection, analysis, interpretation of data, and the writing of this article or the decision to submit it for publication.

## Conflict of Interest

S-KC was employed by Excelsior Biopharma Inc. The remaining authors declare that the research was conducted in the absence of any commercial or financial relationships that could be construed as a potential conflict of interest.

## Publisher's Note

All claims expressed in this article are solely those of the authors and do not necessarily represent those of their affiliated organizations, or those of the publisher, the editors and the reviewers. Any product that may be evaluated in this article, or claim that may be made by its manufacturer, is not guaranteed or endorsed by the publisher.

## Supplementary Material

Supplementary Figure 1 The result of CNVs on *BMPR2* gene of pulmonary arterial hypertension (PAH) patient A450 by multiplex ligation-dependent probe amplification (MLPA) technology. **(A)** The probe signals indicated that there was a heterozygous deletion from *BMPR2* exon 8 to exon 9 in PAH patient A450. **(B)** The quantification plot of copy number variation of A450 on *BMPR2, ENG*, and *ACVRL1* genes using the SALSA MLPA Probemix P093 HHT/HPAH kit (MRC-Holland, Netherlands). BMPR2 5: the sample analytical number of A450. WT: the sample derived from normal subjects.

Supplementary Table 1 Details of genetic variants in *EIF2AK4* (NM_001013703.4).
