# Supplementary figures and images for: Whole Exome Sequencing of Patients With Heritable and Idiopathic Pulmonary Arterial Hypertension in Central Taiwan
Source: Front Cardiovasc Med. 2022 Jun 22;9:911649. doi: 10.3389/fcvm.2022.911649 (PMC9256950; doi:10.3389/fcvm.2022.911649)

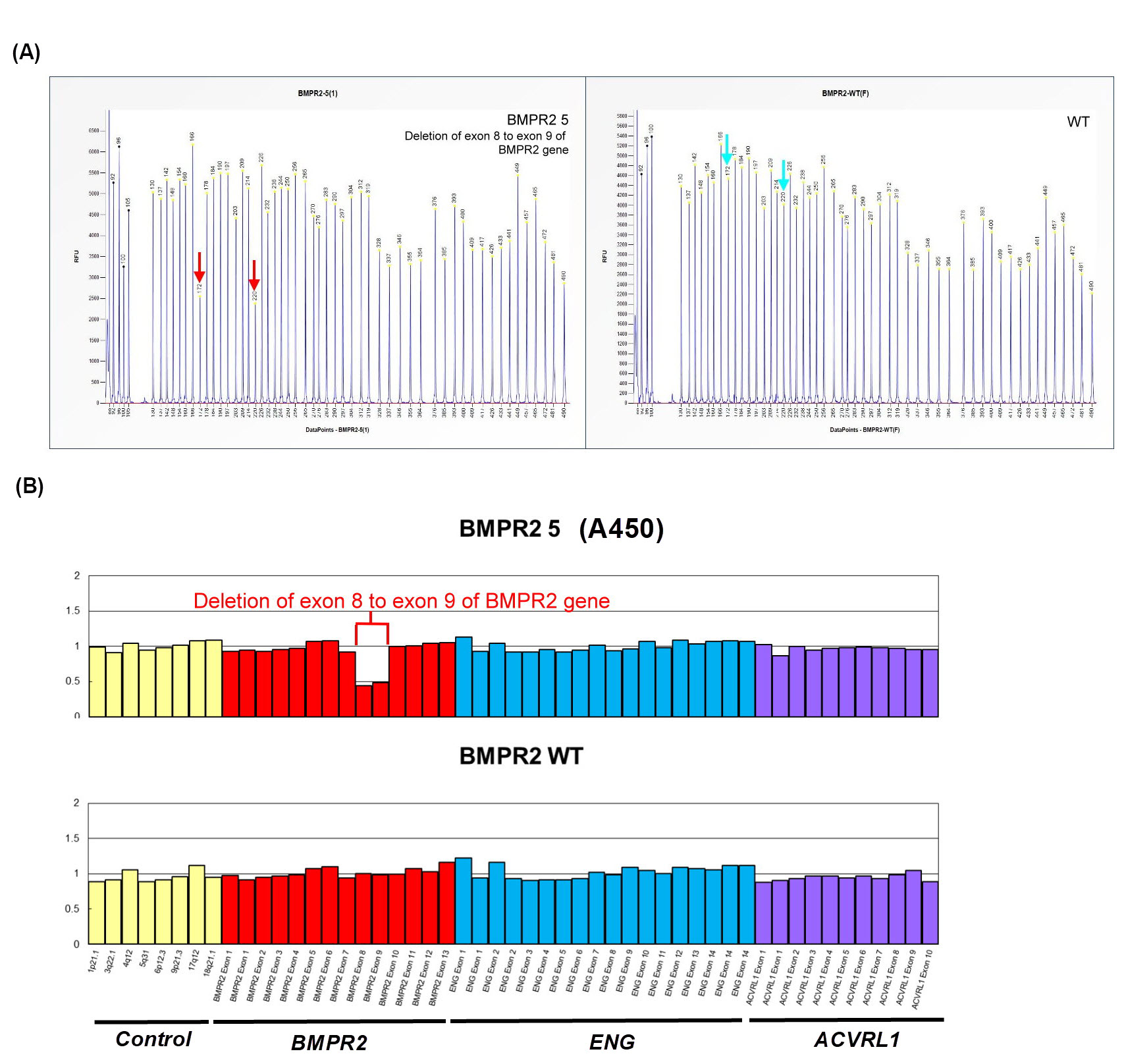

Supplement: Supplementary Figure 1 — The result of CNVs on BMPR2 gene of pulmonary arterial hypertension (PAH) patient A450 by multiplex ligation-dependent probe amplification (MLPA) technology. (A) The probe signals indicated that there was a heterozygous deletion from BMPR2 exon 8 to exon 9 in PAH patient A450. (B) The quantification plot of copy number variation of A450 on BMPR2, ENG, and ACVRL1 genes using the SALSA MLPA Probemix P093 HHT/HPAH kit (MRC-Holland, Netherlands). BMPR2 5: the sample analytical number of A450. WT: the sample derived from normal subjects. [file Image_1.JPEG]
