# Supplementary material for: Whole Exome Sequencing of Patients With Heritable and Idiopathic Pulmonary Arterial Hypertension in Central Taiwan
Source: Front Cardiovasc Med. 2022 Jun 22;9:911649. doi: 10.3389/fcvm.2022.911649 (PMC9256950; doi:10.3389/fcvm.2022.911649)
Supplement: Supplementary Table 1 — Details of genetic variants in EIF2AK4 (NM_001013703.4). [file Table_1.doc]

|  | **Supplement Table 1. Details of genetic variants in *EIF2AK4* (NM_001013703.4)** | | | | | | | | | | | |
| --- | --- | --- | --- | --- | --- | --- | --- | --- | --- | --- | --- | --- |
| **No.** | **ID** | **Gender** | **WSPH** | **Gene** | **Nucleotide change (cDNA)** | **Amino acid change** | **Variant type** | **Genotype** | **PolyPhen2 /SIFT** | **ACMG** | **MAF in EAS** | **Ref** |
|  |  |  | **genes** |  |  |  |  |  |  | **2015** |  |  |
| **1** | A212 | F | Y | *EIF2AK4* | c.2464A>G | p.Thr822Ala | Missense | Het | B/T | VUS | 0.00044 | - |
| **2** | A489 | F | Y | *EIF2AK4* | c.1037A>G | p.Glu346Gly | Missense | Het | PD/D | VUS | 0.00000 | - |
| **3** | A522 | F | Y | *EIF2AK4* | c.626A>G | p.Gln209Arg | Missense | Het | PD/D | VUS | 0.00073 | - |

WSPH: The 6th World Symposium on Pulmonary Hypertension; ACMG: 2015 The American College of Medical Genetics and Genomics guidelines; MAF: Minor allele frequency of East Asian in gnomAD exome databases; PolyPhen2: PD, Probably damaging; D, Damaging; B, Benign. SIFT: D, Deleterious, T, Tolerated.
